# Transcriptional comparison of Testicular Adrenal Rest Tumors with fetal and adult tissues

**DOI:** 10.1530/EJE-22-0143

**Published:** 2022-09-29

**Authors:** Mariska A.M. Schröder, Fred C.G.J. Sweep, Antonius E. van Herwaarden, Rod T. Mitchell, Jitske Eliveld, Ans M. M. van Pelt, Alan E. Rowan, Darren Korbie, Nike M.M.L. Stikkelbroeck, Hedi L. Claahsen - van der Grinten, Paul N. Span

**Affiliations:** 1 Department of Pediatrics, Radboud Amalia Children’s Hospital, Radboud university medical center, 6500HB Nijmegen, The Netherlands; 2 Department of Laboratory Medicine, Radboud Institute for Molecular Life Sciences (RIMLS), Radboud university medical center, 6500HB Nijmegen, The Netherlands; 3 MRC Centre for Reproductive Health, University of Edinburgh, Edinburgh EH16 4TJ, United Kingdom; 4 Center for Reproductive Medicine, Reproductive Biology Laboratory, Amsterdam Reproduction and Development Research Institute, Amsterdam UMC, University of Amsterdam, 1105 AZ, Amsterdam, the Netherlands; 5 Australian Institute for Bioengineering and Nanotechnology, The University of Queensland, Brisbane Qld 4072, Australia; 6 Department of Internal Medicine, Radboud University Medical Center, 6500 HB Nijmegen, The Netherlands; 7 Radiotherapy & OncoImmunology Laboratory, Department of Radiation Oncology, Radboud Institute for Molecular Life Sciences (RIMLS), Radboud university medical center, 6500HB Nijmegen, The Netherlands

## Abstract

**Background:**

Testicular Adrenal Rest Tumors (TART) are a common complication of unknown cellular origin in patients with Congenital Adrenal Hyperplasia (CAH). These benign tumors have both adrenal and testicular characteristics and are hypothesized to either derive from cells of adrenal origin from the fetal adrenogonadal primordium or by atypical differentiation of adult Leydig-progenitor cells.

**Objective:**

This study aims to unravel the identity and etiology of TART.

**Methods:**

Co-expression of adrenal-specific CYP11B1 and Leydig cell-specific HSD17B3 in TART was studied using immunohistochemistry. We studied the possibility of TART being derived from atypical differentiation of adult Leydig-progenitor cells by the quantification of adrenal-specific enzyme expression upon ACTH-like stimulation of ex vivo cultured PDGFRA-positive cells. By comparing the transcriptome of TART (n=16) with the transcriptome of fetal adrenal (n=13), fetal testis (n=5), adult adrenal (n=11) and adult testis (n=10) tissues, we explored the identity of TART.

**Results:**

We demonstrate co-expression of adrenal-specific CYP11B1 and testis-specific HSD17B3 in TART cells, indicating the existence of a distinct TART cell exhibiting both adrenal and testicular characteristics. *Ex vivo* cultured adult Leydig-progenitor cells did not express the ACTH-receptor *MC2R* but did express *CYP11B1* upon stimulation. Unsupervised clustering of transcriptome data showed that TART was most similar to adult adrenal tissue, followed by adult testis tissue, and least similar to either fetal tissue.

**Conclusion:**

Our data suggest that TART is induced -most likely via activation of a cAMP/PKA dependent receptor-from a progenitor cell into a unique mature adrenal-like cell type, sometimes exhibiting both adrenal and testicular features.

## Introduction

Congenital Adrenal Hyperplasia (CAH), often caused by defects in steroid 21-hydroxylase (CYP21A2), is an autosomal recessive disorder affecting adrenal steroid biosynthesis, leading to impaired glucocorticoid production (1). Consequently, due to a lack of negative feedback by glucocorticoids, adrenocorticotropin hormone (ACTH) production by the pituitary gland is increased, resulting in hyperplasia of the adrenal cortex (2) and ACTH-driven steroid hormone biosynthesis via the activation of cAMP/protein kinase A signaling. Steroid precursors upstream of the enzymatic defect accumulate and shunt into the non-affected adrenal androgen pathway with consequently elevated adrenal androgen production (2). CAH-associated benign nodules in the testis are a common complication (prevalence ranging from 14-89%) in male CAH patients (3) and are of concern as they may cause testicular damage and infertility. The tumors have histological and functional features of adrenal tissue (4) and are referred to as ‘testicular adrenal rest tumors’ (TART). However, the origin and etiological features of TART are still unclear, impeding prevention and treatment.

Initially, TART was considered to originate from ectopic embryologic adrenal rests that descend along with the gonads during embryological development. Chronically elevated ACTH levels present *in utero* may prevent the regression of this ectopic adrenal tissue. Clark et al. (5) reported that TART tissue is homogeneous with morphological and biochemical characteristics of steroid-secreting adrenocortical cells with little similarity to normal testicular tissue. However, in addition to the expression of genes for adrenal-specific enzymes (*CYP11B1* and *CYP11B2*) and receptors (ACTH receptor (*MC2R*); Angiotensin II receptor), testicular-specific gene expression patterns (*HSD17B3; LHCGR; GATA4*; possibly *INSL3*) have also been reported for TART (3). The combination of adrenal- and testis-specific markers might indicate that TART arises from a common undifferentiated pluripotent progenitor, as the testis and adrenal gland are derived from the same adrenogonadal primordium. One of the candidate cell types are fetal Leydig cells (FLC), which are reported to be ACTH-sensitive (6). TART appears to be already present in neonates (7) and infants (8), supporting the hypothesis of an embryological origin of TART. Val et al. (9) described the presence of an alternative adrenal-like cell type in the normal-developing and adult mouse testis, which presents both adrenal and Leydig-cell properties (hCG responsiveness) and suggests that testicular adrenal rests may develop from this cell population. Benvenga et al. (10) hypothesized that pluripotent ACTH-sensitive cells in the testis may differentiate into either an adrenal cell type or testicular cell type depending on their stimulus.

Multiple studies (11–13) have focused on the characterization of TART using an a priori selection of markers, but an unbiased characterization of TART is lacking. This study aims to unravel the identity and etiology of TART, to facilitate future prevention and improve management of TART, by comparing the transcriptomic profiles of TART with both fetal and adult testis and adrenal tissues. By including both fetal and adult adrenal and testis tissues in the (targeted) RNA sequencing panel, we aim to establish whether TART has a multipotent progenitor phenotype or a more differentiated mature phenotype.

## Materials and Methods

### Immunohistochemistry

Double immunofluorescence staining was performed on formalin-fixed paraffin-embedded material of TART, adult testis and adult adrenal tissue, obtained from the pathology department (Radboudumc). Sections were deparaffinized and hydrated and antigen retrieval was performed in Tris-EDTA buffer (pH 9) at 95°C for 30 minutes. Samples were blocked for 30 minutes with 5% normal donkey serum and incubated overnight with adrenal-specific monoclonal CYP11B1 (hCYP11B1-80-2-2, kindly provided by Prof. Dr. Gomez-Sanchez (14); 1:200) and Leydig cell-specific HSD17B3 (13415-1-AP; Proteintech; 1:100). Samples were incubated for 90 minutes with Alexa Fluor 488-conjugated donkey-anti-rabbit secondary antibody (A21206; Invitrogen; 1:300) and Cyanine Cy™3-conjugated donkey-anti-rat secondary antibody (712-166-150; Jackson ImmunoResearch; 1:300) at room temperature. Slides were counterstained for 5 minutes with DAPI (1:500). Tissue sections were imaged at 630x on a Zeiss LSM900 using the Zeiss Zen 3.0 software. Brightness was equally adjusted. For overview images, slides were incubated with 0.25% Sudan Black B in 70% ethanol, washed three times with PBS, counterstained for 5 minutes with Hoechst (1:3000) and imaged at 200x on a Zeiss Axio Scope A1.

### Expression of adrenal-specific genes in PDGFRA-positive testicular cells

Levels of *MC2R, AGTR2, CYP11B1, and CYP21A2* were quantified in previously isolated RNA from *ex vivo* cultured human testicular multipotent PDGFRA-positive cells stimulated for 24h with dbcAMP, forskolin, hCG, or basal media (15). For this, testicular interstitial cells were isolated from two donors, expanded in vitro and FACS sorted for PDGFRA-positive cells. These PDGFRA-positive cells, which include the adult stem Leydig cells and adult progenitor Leydig cells (here referred to as adult progenitor Leydig cells), were further expanded for 28 days prior to this 24h treatment stimulation period, as described in detail elsewhere (15). For qPCR, RNA was isolated using the PicoPure RNA isolation kit (Arcturus, Thermo Fisher Scientific), followed by two times on-column DNase treatment (Qiagen, Germany). As positive and negative controls, RNA isolated (as described below) from adult/fetal adrenal and adult testis were included. Isolated RNA was reversed transcribed using SuperScript IV First-Strand Synthesis System (Invitrogen) and Biometra Thermocycler after annealing random hexamers to the RNA (5 minutes at 65 °C). PrimePCR™ SYBR® Green Assays for *CYP21A2, CYP11B1*, and *AGTR2* (BioRad) and self-designed primers for *MC2R* (Forward: CAGAGCTGAAGGTGATTGGGA; Reverse: AAGGCGGGGATGTTACTTGG) and housekeeping genes *PBGD* and *GAPDH* were used. Gene expression was quantified in a CFX96 Touch Real-Time PCR Detection System (Bio-Rad), after combining 4 μL ten-times diluted cDNA with 1 μL of primer assay or 1.2 μL forward and reverse primer, 10 μL FastStart SYBR Green Master (Roche Diagnostics), and 5 μL or 3.6 μL MilliQ. Expression levels relative to the housekeeping genes were quantified using the delta CT method. No quantified expression (Ct>40) was set to zero.

### Tissue collection

In total, sixteen TART samples were used for this study. Twelve bilateral TART samples from six CAH patients (mean age 27 year; range 23-32 year) were collected as previously described (13). Written informed consent was obtained from all patients. Another two frozen histologically proven TART samples from one anonymous CAH patient were obtained and two bilateral TART samples were collected from a 27-year-old patient with recurrent Cushing’s Disease since the age of eleven (16). Those tumors have previously been characterized and showed similar characteristics to TART from CAH patients but were analyzed separately from the TART samples obtained from CAH patients. Frozen anonymous samples of eleven normal adult adrenal glands and ten normal adult testes were collected from the Pathology and Urology departments of our hospital (Radboudumc) and used in accordance with the Code of Conduct of the Federation of Medical Scientific Societies in the Netherlands (http://www.federa.org/codes-conduct). The study was approved by the institutional review board (CMO Radboudumc #2016-2977 and CMO-nr 2004/007). In addition, five fetal testis samples and thirteen fetal adrenal samples (first and second trimester) were anonymously obtained following elective termination of pregnancy from the MRC Centre for Reproductive Health of the University of Edinburgh. Informed consent was obtained with ethical approval from the South East Scotland Research Ethics Committee (LREC08/S1101/1). The study conforms to the principles set out in the WMA Declaration of Helsinki.

### RNA isolation, library preparation, and sequencing

RNA was isolated from frozen tissue samples using the total RNA Purification Kit (Norgen Biotek) according to the manufacturer’s protocol. RNA quantity and purity were assessed using a NanoDrop 2000 Spectrophotometer. RNA was reversed transcribed using Superscript VILO cDNA synthesis kit (Thermo Fisher Scientific) using a C1000 Thermal cycler (Biorad), according to the manufacturer’s protocol. Libraries were constructed using the Ion AmpliSeq Transcriptome Human Gene Expression kit (Thermofisher). Templating and sequencing were performed with the Ion PI HiQ OT200 kit, and Ion PI HiQ sequencing kit using Ion PI V3 chips (Life Technologies) and a Proton Sequencer (Thermofisher). All RNA samples gave libraries of the expected size, with no observable amplification of no-template controls. Mapping of read data was performed automatically on the Torrent Suite server, against a reference fasta file composed of the transcript sequences of the target genes, and total read counts per target gene were then tallied.

### Unsupervised clustering analysis

Read counts were normalized using the R/Bioconductor package DESeq2 (R version 3.6.2) (17), which normalizes for both sequencing depth and RNA composition. The absence of batch effects was confirmed using Principal Component Analysis (PCA). Unsupervised hierarchical clustering (clustering method ‘complete’) was performed, after logarithmic transformation using the Variance Stabilizing Transformation function (DESeq2). PCA was performed on transformed data of the top 500 genes with the highest variance over the different samples.

### Differential expression analysis

Differential expression analysis on the transcriptome data was performed using DESeq2. The threshold for differentially expressed genes was set at an absolute fold-change > 2. P-values were adjusted by the Benjamini-Hochberg method to correct for multiple testing and control for false-discovery rate (FDR). Adjusted p-values < 0.05 were considered statistically significant.

## Results

### Adrenal- and Leydig cell-specific proteins co-localize in TART cells

**Figure 1:**
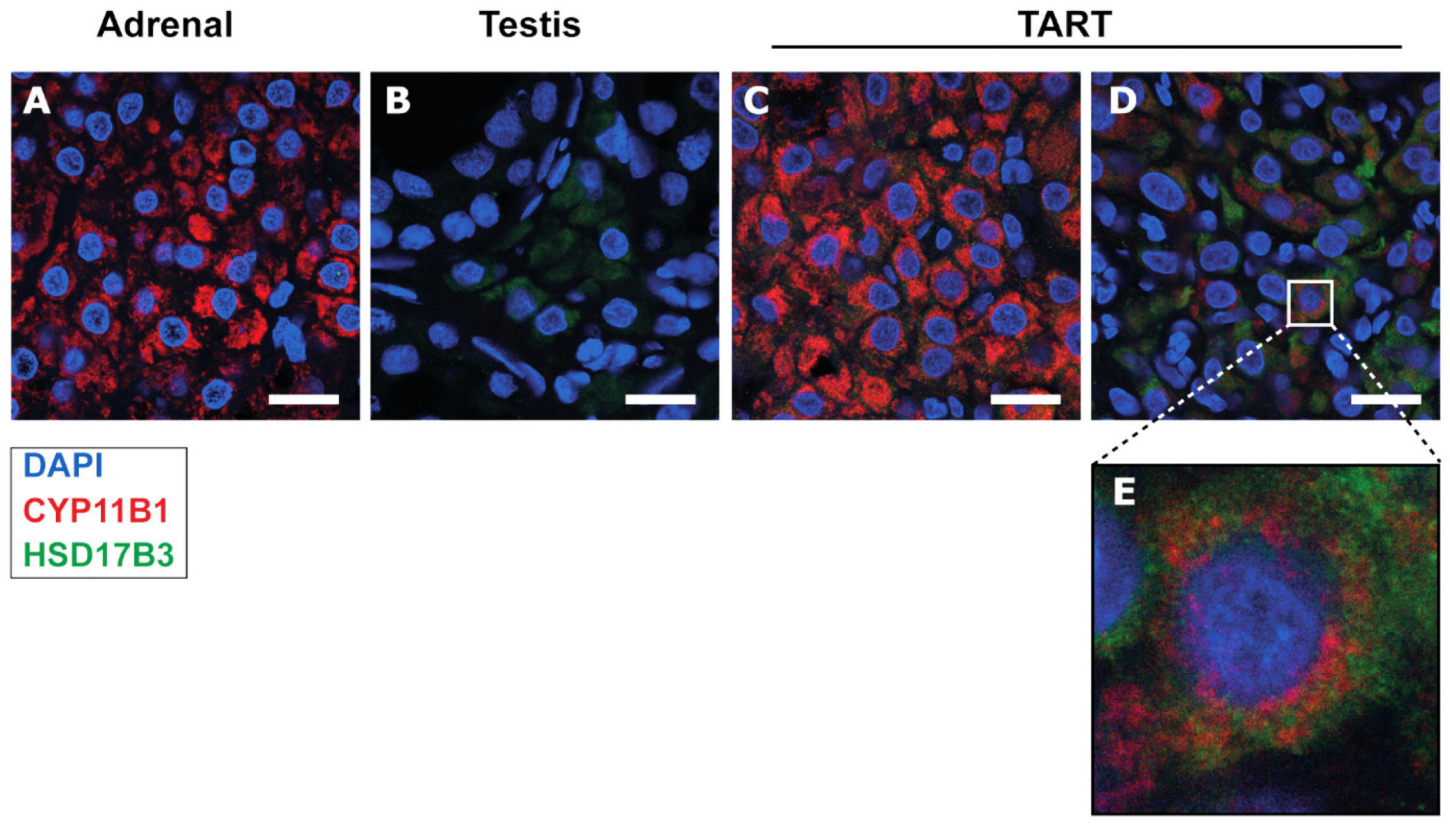
Co-localization of CYP11B1 and HSD17B3 in TART. Double immunohistochemical staining of adrenal-specific 11β-hydroxylase (CYP11B1, red) and Leydig cell-specific l7β-hydroxysteroid dehydrogenase 3 (HSD17B3, green) on formaldehyde-fixed-paraffin-embedded (FFPE) material of adult adrenal, adult testis, and TART tissues. Normal adult adrenal tissue (HSD17B3-/CYP11B1+) and adult testis tissue (HSD17B3+/CYP11B1-) served as positive and as negative controls. Scale bar indicates 20 μm.

Spatial localization of adrenal-specific Cytochrome P450 11B1 (CYP11B1) and Leydig cell-specific 17beta-Hydroxysteroid dehydrogenase 3 (HSD17B3) was studied at the protein level using immunohistochemistry (Figure 1). Most TART cells expressed adrenal-specific CYP11B1 (Figure 1 C), and testis-specific HSD17B3 (18) expression was observed in a smaller subset of TART cells (Supplementary Figure 1); Overall, approximately 25% of the CYP11B1-positive cells also expressed HSD17B3. Intriguingly, when present in TART tissue, the HSD17B3 staining co-localized with the CYP11B1 staining within the same cells (Figure 1E; Supplementary Figure 1). Thus, the mixed adrenal and testis-like features of TART we described earlier (3) are not due to a mixture of adrenal and testis-like cells but is -at least partly-associated with co-occurrence of both features within a single cell type.

### Adult progenitor Leydig cells do not express MC2R

**Figure 2:**
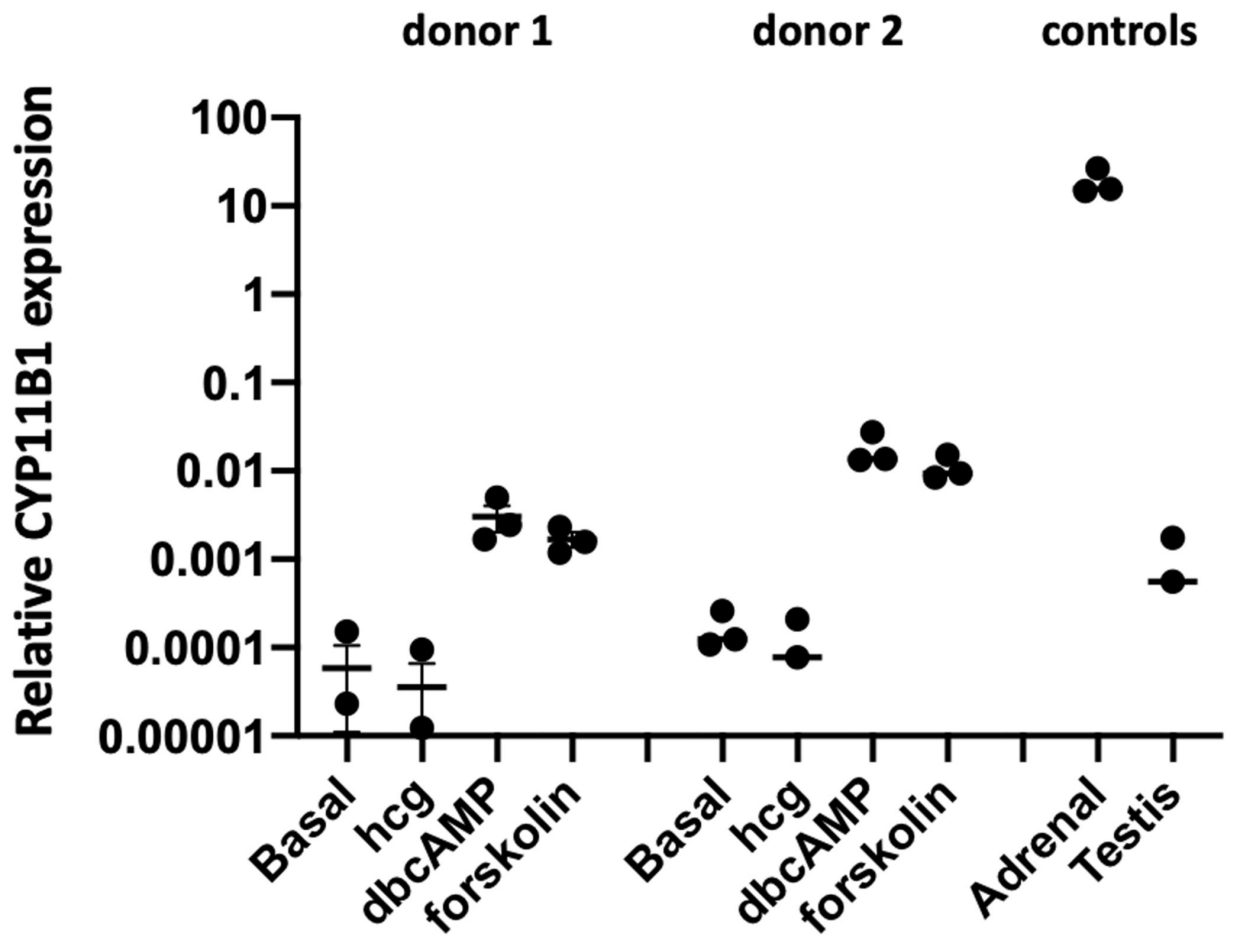
CYP11B1 expression in ACTH-like treated ex vivo cultured human PDGFRA-positive cells Relative *CYP11B1* expression in ex vivo cultured PDGFRA-positive testicular cells isolated from two donors, treated with basal media, hCG, dbcAMP, or forskolin. RNA isolated from healthy adult adrenal or healthy adult testis tissue were included as positive and negative controls. Expression relative to the geometric mean expression of the reference genes *PBGD* and *GAPDH* was determined using the delta Ct method.

To study the possibility of TART being derived from atypical differentiation of adult progenitor Leydig cells, we verified if Platelet Derived Growth Factor Receptor Alpha (PDGFRA)-positive human interstitial testicular cells, which include the adult stem and progenitor Leydig cells, express the ACTH receptor (*MC2R*) or the Angiotensin II receptor (*AGTR2*), and whether these cells express adrenal enzymes *CYP11B1* and *CYP21A2* after stimulation with forskolin or dbcAMP. Forskolin and dbcAMP are known to raise the intracellular cAMP levels and activate the protein kinase A (PKA) pathway, which regulates the expression of steroidogenic enzymes. No expression of *MC2R, AGTR2*, or *CYP21A2* was detected in the propagated testicular PDGFRA-positive cells, in any of the conditions (data now shown). Expression of *MC2R, AGTR2*, and *CYP21A2* were validated in adult adrenal tissue (positive control) or fetal adrenal tissue (*AGTR2*). *CYP11B1* expression was detected in PDGFRA-positive cells, and dbcAMP and forskolin treatment resulted in elevated levels of its expression in both donors compared to basal conditions, although expression was low in comparison to untreated adult adrenal tissue (Figure 2). Upon dbcAMP or forskolin treatment of PDGFRA-positive cells, *CYP11B1* expression exceeded the low level of expression in untreated adult testis tissue, while hCG had no effect on *CYP11B1* expression. Thus, while *CYP11B1* expression is induced in two PDGFRA-positive samples, this induction is not mediated via MC2R but another receptor using cAMP and PKA as second messenger.

### Transcriptome of TART is most similar to adult tissues

**Figure 3:**
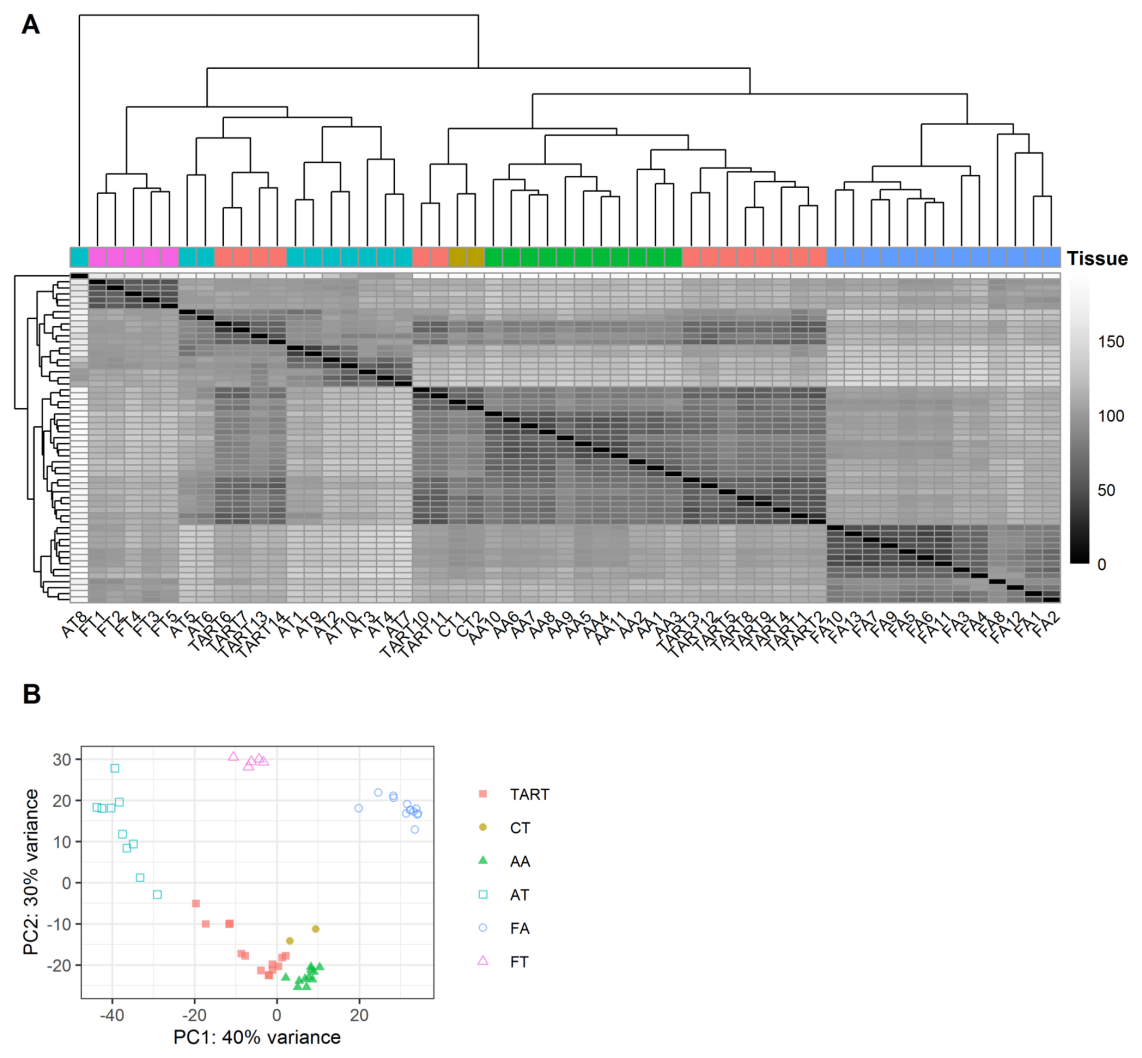
Similarity of TART and reference tissues Hierarchical clustering analysis (A) and Principal Component Analysis (B) of TART and reference tissues. Abbreviations: CT = Cushing-TART; AA = Adult Adrenal; AT = Adult Testis; FA = Fetal Adrenal; FT = Fetal Testis. PC1 = Principal Component 1; PC2 = Principal Component 2.

Alternatively, TART may arise from fetal adrenogonadal primordium cells. In order to study the (dis)similarity of TART with adult adrenal-, adult testis-, fetal adrenal-, and fetal testis tissues, targeted transcriptome sequencing was performed. Principal component analysis (PCA) revealed that the different tissues were well clustered and effectively separated based on high variance genes (Figure 3B). Both PCA and unsupervised hierarchical clustering showed that TART was most similar to either adult (adrenal- and testis) tissue, and least similar to either fetal (adrenal and testis) tissue (Figure 3A/B). Minimal deviation was identified between bilateral TART samples obtained from the same patient, indicating minimal intra-patient heterogeneity. There was some variation between TART, where some TART samples showed less variance with regard to adult testis samples than other TART samples. The four TART samples (TART6,7,13,14) that were clustered together with adult testis tissue by hierarchical clustering (figure 3A) showed least variance with regard to the testis samples compared to the other TART samples in the PCA plot (figure 3B). Thus, the transcriptome of TART was most similar to adult adrenal tissue followed by adult testis tissue and least similar to fetal adrenal and fetal testis tissue.

### TART does not express fetal genes

**Figure 4:**
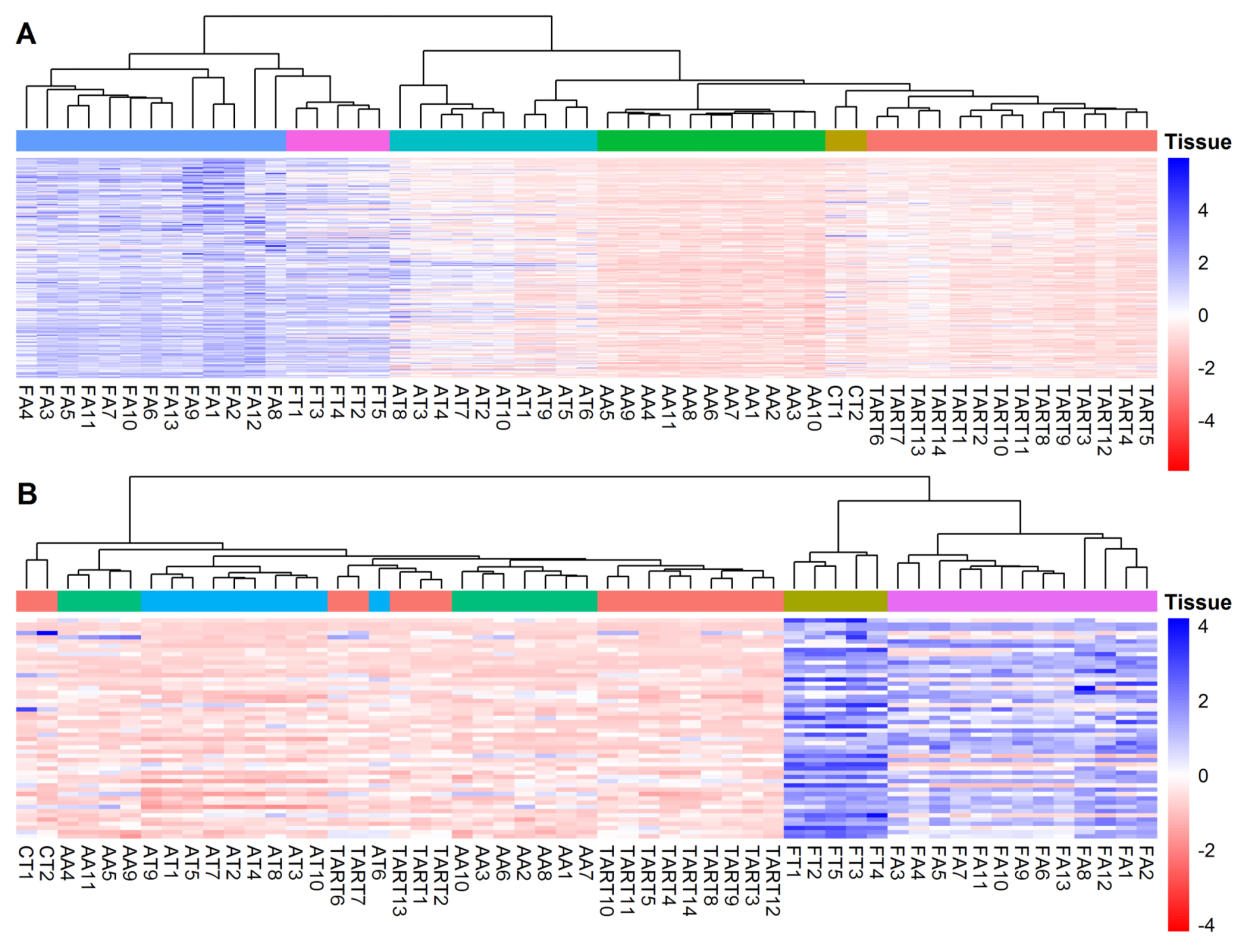
Relative expression of fetal-signature genes by TART Heatmap showing the relative expression of significantly upregulated genes (fold-change > 2) in fetal adrenal versus adult adrenal tissue (A) and fetal testis versus adult testis tissue (B) in TART, Cushing-TART (CT), Adult Adrenal (AA), Adult Testis (AT), Fetal Adrenal (FA), and Fetal Testis (FT) samples. Blue color indicates relatively higher expression and red color indicates relatively lower expression compared to average expression.

To examine the expression of fetal-signature genes, we visualized the expression of genes that were significantly upregulated (fold-change > 2) in fetal adrenal versus adult adrenal tissue (Figure 4A) and fetal testis versus adult testis tissue (Figure 4B). The resulting heatmaps demonstrated that, overall, TART expressed these fetal signature genes at similar levels as the adult tissues.

### TART express both adrenal- and testis-specific genes

**Figure 5:**
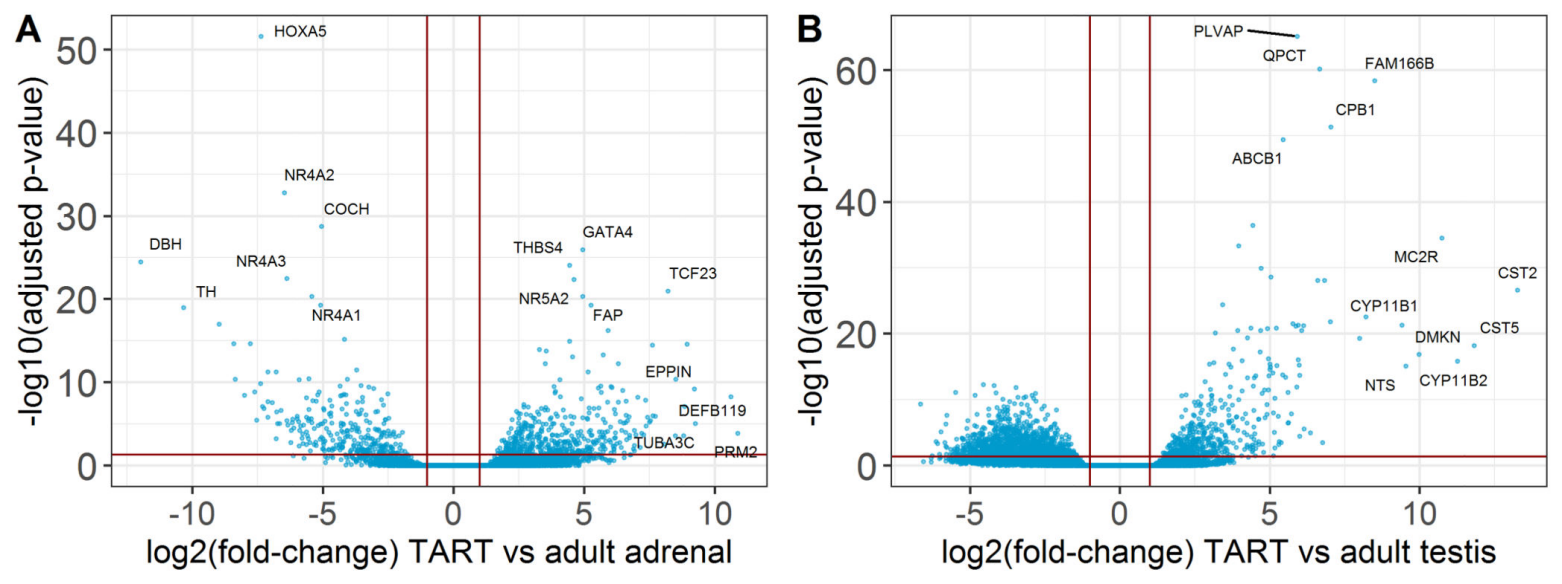
Differentially expressed genes in TART. Volcano plots of differentially expressed genes in TART compared to adult testis tissue (A) and adult adrenal tissue (B). Vertical red lines indicate the log2 fold-change threshold at -1 and 1 and the horizontal line indicates the adjusted p-value threshold at 0.05.

To explore TART-specific gene expression patterns, the transcriptomic profile of TART was compared with the transcriptomic profile of adult testis and adrenal tissues. Differential expression analysis identified 1877 differentially expressed genes (DEGs) (fold-change > 2; adjusted p-value < 0.05) in TART compared to adult testis tissue (494 upregulated; 1383 downregulated) (Figure 5A). TART overexpressed the adrenal-specific genes *CYP11B1, CYP11B2*, and *MC2R* compared to testis tissue. Compared to adult adrenal tissue, 728 genes were differentially expressed in TART (449 upregulated; 279 downregulated) (Figure 5B). TART highly overexpressed testis-specific *DEFB119, PRM2*, and *GATA4* (Figure 5A) compared to adrenal tissue. According to the Human Protein Atlas, 429 genes are expressed specifically in the testis (more than four-fold higher expressed compared to other analyzed tissues). At least 31 of those testis-specific genes were identified to be more highly expressed in TART compared to adrenal tissue. Surprisingly, both Leydig cell-specific- (*INSL3; HSD17B3*) as well as germ-cell specific genes (e.g. *FATE1; DDX4*) (19–22) were overexpressed in TART compared to adult adrenal tissue, especially in the TART samples that deviated most from adrenal tissue with PCA (TART 6,7,12,13,14). The ACTH receptor (*MC2R*) and Angiotensin II receptor were overexpressed in TART versus adult testis tissue. Only very low numbers of normalized read counts for the *LHCGR* receptor in TART samples and no significant overexpression of the LHCG receptor in TART compared to adult adrenal tissue was found, as the adrenal gland showed similarly low levels of *LHCGR* expression (data not shown).

TART does express GLI family zinc finger 1 (*GLI1*), Wilms tumor protein homolog 1 (*WT1*), and Nestin (*NES;* data not shown), which are genes that are preferentially expressed by undifferentiated non-steroidogenic adrenocortical cells (23–25).Overall, the transcriptomic data do not support a fetal adrenogonadal primordium-like phenotype of TART.

## Discussion

This study aimed to investigate the etiology of TART. Because of previous identification of both adrenal and testicular markers in TART (3), we speculated that TART might be derived from a more multipotent progenitor cell type such as fetal Leydig progenitor cells or adult progenitor Leydig cells. Indeed, here we show that TART cells contain both adrenal and Leydig cell characteristics, and thus constitute a unique adreno-testicular phenotype. We subsequently demonstrated that isolated PDGFRA-positive testicular interstitial cells, which include adult stem and progenitor Leydig cells, do not express the ACTH-receptor *MC2R*. These *ex-vivo* cultured adult progenitor Leydig cells did not exhibit adrenal-like expression patterns, but upon forskolin- or dbcAMP- (ACTH-like) stimulation *CYP11B1* expression was elevated. Thus, another receptor than MC2R, using cAMP and PKA as second messenger, may induce differentiation of adult progenitor Leydig cells into a distinct cell type that expresses both adrenal- and testis-specific markers. As the TART transcriptome was least similar to fetal adrenal and testis tissues, a ‘fetal’ progenitor-like phenotype of TART is unlikely.

We present the most robust characterization of TART to date, where the transcriptome of fourteen bilateral TART samples from seven CAH patients and two bilateral TART samples from a patient with recurrent Cushing’s disease was compared with adult adrenal samples (n=11), adult testis samples (n=10), fetal adrenal samples (n=13), and fetal testis samples (n=5). The transcriptome of TART was most similar to adult adrenal samples, followed by adult testis samples, suggesting that TART cells have differentiated into a mature and unique distinctive cell type. Although we here find that the transcriptome of TART tissues is clearly distinct from fetal tissues, we did not prove that TART does not originate from fetal Leydig cells. TART being derived from a fetal progenitor cell is still possible as we initially hypothesized, given the fact that TART is likely already present *in utero* and its resemblance to both testis and adrenal tissues which derive from a common primordium. However, in an endeavor to find the cell of origin of TART we searched for *MC2R*-positive cells in publicly available single-cell datasets of fetal (GSE143356 (26)), neonatal (GSE124263 (27)), and adult testes (GSE124263 (27)). No *MC2R*-positive cell populations were identified in these healthy testes, what may suggest that TART arises from cell types that are not typically present in the healthy testis (i.e. adrenal rests). Alternatively, it could suggest that other (melanocortin) receptors play a role in the hyperplasia of these cells that are responsive to ACTH or that other factors are required for TART development. While it has been described that ACTH can stimulate testicular steroidogenesis during fetal development in mice (28), this has not been demonstrated for the human fetal testis. Further research is required to evaluate ACTH-responsiveness of the human fetal testis.

Our study emphasizes the high similarity between TART and adult adrenal tissue and highlighted the adrenal steroidogenic properties of TART. The marked increased expression of the adrenal-specific genes *CYP11B1, CYP11B2* and *MC2R* in TART compared to testis tissue is in line with our previous research where we showed the expression of those genes in the TART samples using RT-qPCR, and underscores the robustness of our study (13). G*ATA4* expression in TART has also been reported previously by our group (29). ACTH and angiotensin II have trophic effects on adrenocortical tissue (30, 31), and likely play a major role in the growth of TART (5, 11). Previous research by other groups proposed that the LH/hCG receptor (LHCGR) might play a role in the etiology of TART(10, 32). However, our data showed equally low LHCGR expression in TART samples as adult adrenal tissues, which is consistent with previous findings, where we reported nearly equal expression levels of *LHCGR* in TART and adrenal tissue (11). Adrenocortical expression of low levels of *LHCGR* has been reported previously (33).

It is unknown if an undifferentiated progenitor cell resides within TART that replenishes steroidogenic TART cells. This RNAseq dataset of TART shows that TART expresses genes (GLI1, WT1, NES) that are preferentially expressed by undifferentiated non-steroidogenic adrenocortical cells (23–25). Further research is needed to identify and characterize a potentially present non-steroidogenic progenitor/stem cell pool within TART.Our study also demonstrated the expression of testis-specific genes in TART samples in addition to the adrenal specific gene expression. The co-expression of adrenal and testicular genes in TART is in agreement with previous findings of our group and a recent study by Kolli and colleagues (34). With PCA, TART samples were positioned between the adrenal tissue samples and testis tissue samples. Remarkably, in addition to upregulation of genes specific for somatic testicular cells, we also unexpectedly identified upregulation of genes specifically expressed in germ cells. Because of the expression of both germ cell-specific genes and Leydig cell-specific genes, it could be argued that the expression of those testis-specific genes in TART is caused by the presence of surrounding testis tissue in the TART biopsies or that TART cells are intermingled with healthy Leydig and germ cells. However, the TART tissues removed during surgery were well-demarcated. Although complete absence of mature Leydig cells in TART cannot be guaranteed, the co-expression of adrenalspecific CYP11B1 and testis-specific HSD17B3 in TART tissue indicated the presence of a distinct and unique cell type with both adrenal- and testis-specific features. It should be mentioned that, whereas CYP11B1 was expressed in nearly all cells, HSD17B3 was only expressed in small subsets of cells. We observed some variation between individual TART samples, as some TART samples were more similar to testis tissue and presented higher expression of testis-specific genes compared to other TART samples. The variation between TART samples, and more specifically between the TART samples of different patients, might explain the contradicting results of previous case reports where TART with (3) or without (5) testis-specific characteristics have been reported.

We do acknowledge several limitations in this study. Firstly, adrenal medullary RNA was present in the sequenced adrenal samples, while TART samples do not contain adrenomedullary cells. The adrenal medulla contains transformed chromaffinergic cells and the presence of these cells in the adrenal tissue, as well as the presence of other testicular cells than Leydig cells in the testis samples, likely has affected our analysis. For this reason, single cell approaches are of interest for future studies, which also allow to study the heterogeneity of TART. Secondly, while the expression of adrenal-specific genes by isolated PDGFRA-positive testicular cells upon dbcAMP or forskolin treatment is insightful, we were unable to identify the receptor responsible for this.

In conclusion, this study presents the most robust molecular characterization of TART to date; We report that TART is most similar to adult adrenal tissue, followed by adult testis tissue, and least similar to the fetal adrenal and testis tissues, suggesting that TART has differentiated into a mature adrenal-like cell and partially in a mature and distinct unique cell type, exhibiting both adrenal and testicular features.

## Supplementary Material

Supplementary Figure 1 Overview of two TART tissues (co-)expressing adrenal-specific 11β-hydroxylase (CYP11B1, red) and Leydig cell-specific 17β-hydroxysteroid dehydrogenase 3 (HSD17B3, green). Nuclei were stained with Hoechst (blue).

## Data Availability

The RNA-sequencing dataset is deposited in the gene expression omnibus (GSE196700).

## Abbreviations

ACTH: Adrenocorticotropic hormone
CAH: Congenital Adrenal Hyperplasia
CYP11B1: Cytochrome P450 11B1
HSD17B3: 17beta-Hydroxysteroid dehydrogenase 3
MC2R: Melanocortin 2 Receptor
TART: Testicular Adrenal Rest Tumors

## References

[1] Claahsen-van der Grinten HL, Speiser PW, Ahmed SF, Arlt W, Auchus RJ, Falhammar H, Fluck CE, Guasti L, Huebner A, Kortmann BBM, Krone N (2021). Congenital adrenal hyperplasia - current insights in pathophysiology, diagnostics and management. Endocrine Reviews.

[2] Witchel SF (2017). Congenital Adrenal Hyperplasia. Journal of Pediatric and Adolescent Gynecology.

[3] Engels M, Span PN, van Herwaarden AE, Sweep F, Stikkelbroeck N, Claahsen-van der Grinten HL (2019). Testicular Adrenal Rest Tumors: Current Insights on Prevalence, Characteristics, Origin, and Treatment. Endocrine Reviews.

[4] Claahsen-van der Grinten HL, Otten BJ, Hermus AR, Sweep FC, Hulsbergen-van de Kaa CA (2008). Testicular adrenal rest tumors in patients with congenital adrenal hyperplasia can cause severe testicular damage. Fertility and Sterility.

[5] Clark RV, Albertson BD, Munabi A, Cassorla F, Aguilera G, Warren DW, Sherins RJ, Loriaux DL (1990). Steroidogenic enzyme activities, morphology, and receptor studies of a testicular adrenal rest in a patient with congenital adrenal hyperplasia. Journal of Clinical Endocrinology and Metabolism.

[6] O’Shaughnessy PJ, Baker PJ, Johnston H (2006). The foetal Leydig cell-- differentiation, function and regulation. International Journal of Andrology.

[7] Bouman A, Hulsbergen-van de Kaa C, Claahsen-van der Grinten HL (2011). Prevalence of testicular adrenal rest tissue in neonates. Hormone Research in Paediatrics.

[8] Shanklin DR, Richardson AP, Rothstein G (1963). Testicular Hilar Nodules in Adrenogenital Syndrome. The Nature of the Nodules. American Journal of Diseases of Children.

[9] Val P, Jeays-Ward K, Swain A (2006). Identification of a novel population of adrenal-like cells in the mammalian testis. Developmental Biology.

[10] Benvenga S, Smedile G, Lo Giudice F, Trimarchi F (1999). Testicular adrenal rests: evidence for luteinizing hormone receptors and for distinct types of testicular nodules differing for their autonomization. European Journal of Endocrinology of the European Federation of Endocrine Societies.

[11] Smeets EE, Span PN, van Herwaarden AE, Wevers RA, Hermus AR, Sweep FC, Claahsen-van der Grinten HL (2015). Molecular characterization of testicular adrenal rest tumors in congenital adrenal hyperplasia: lesions with both adrenocortical and Leydig cell features. Journal of Clinical Endocrinology and Metabolism.

[12] Lottrup G, Nielsen JE, Skakkebaek NE, Juul A, Rajpert-De Meyts E (2015). Abundance of DLK1, differential expression of CYP11B1, CYP21A2 and MC2R, and lack of INSL3 distinguish testicular adrenal rest tumours from Leydig cell tumours. European Journal of Endocrinology of the European Federation of Endocrine Societies.

[13] Claahsen-van der Grinten HL, Otten BJ, Sweep FC, Span PN, Ross HA, Meuleman EJ, Hermus AR (2007). Testicular tumors in patients with congenital adrenal hyperplasia due to 21-hydroxylase deficiency show functional features of adrenocortical tissue. Journal of Clinical Endocrinology and Metabolism.

[14] Gomez-Sanchez CE, Qi X, Velarde-Miranda C, Plonczynski MW, Parker CR, Rainey W, Satoh F, Maekawa T, Nakamura Y, Sasano H, Gomez-Sanchez EP (2014). Development of monoclonal antibodies against human CYP11B1 and CYP11B2. Molecular and Cellular Endocrinology.

[15] Eliveld J, van den Berg EA, Chikhovskaya JV, van Daalen SKM, De Winter-Korver CM, van der Veen F, Repping S, Teerds K, van Pelt AMM (2019). Primary human testicular PDGFR alpha(+) cells are multipotent and can be differentiated into cells with Leydig cell characteristics in vitro. Human Reproduction.

[16] Puar T, Engels M, van Herwaarden AE, Sweep FC, Hulsbergen-van de Kaa C, Kamphuis-van Ulzen K, Chortis V, Arlt W, Stikkelbroeck N, Claahsen-van der Grinten HL, Hermus AR (2017). Bilateral Testicular Tumors Resulting in Recurrent Cushing Disease After Bilateral Adrenalectomy. Journal of Clinical Endocrinology and Metabolism.

[17] Love MI, Huber W, Anders S (2014). Moderated estimation of fold change and dispersion for RNA-seq data with DESeq2. Genome Biology.

[18] Geissler WM, Davis DL, Wu L, Bradshaw KD, Patel S, Mendonca BB, Elliston KO, Wilson JD, Russell DW, Andersson S (1994). Male pseudohermaphroditism caused by mutations of testicular 17 beta-hydroxysteroid dehydrogenase 3. Nature Genetics.

[19] Djureinovic D, Fagerberg L, Hallstrom B, Danielsson A, Lindskog C, Uhlen M, Ponten F (2014). The human testis-specific proteome defined by transcriptomics and antibody-based profiling. Molecular Human Reproduction.

[20] Human Protein Atlas http://www.proteinatlas.org.

[21] Kim JY, Jung HJ, Yoon MJ (2015). VASA (DDX4) is a Putative Marker for Spermatogonia, Spermatocytes and Round Spermatids in Stallions. Reproduction in Domestic Animals.

[22] Uhlen M, Fagerberg L, Hallstrom BM, Lindskog C, Oksvold P, Mardinoglu A, Sivertsson A, Kampf C, Sjostedt E, Asplund A, Olsson I (2015). Proteomics. Tissue-based map of the human proteome. Science.

[23] Walczak EM, Hammer GD (2015). Regulation of the adrenocortical stem cell niche: implications for disease. Nature Reviews: Endocrinology.

[24] Lerario AM, Finco I, LaPensee C, Hammer GD (2017). Molecular Mechanisms of Stem/Progenitor Cell Maintenance in the Adrenal Cortex. Frontiers in Endocrinology.

[25] Lyraki R, Schedl A (2021). Adrenal cortex renewal in health and disease. Nature Reviews: Endocrinology.

[26] Chitiashvili T, Dror I, Kim R, Hsu FM, Chaudhari R, Pandolfi E, Chen D, Liebscher S, Schenke-Layland K, Plath K, Clark A (2020). Female human primordial germ cells display X-chromosome dosage compensation despite the absence of X-inactivation. Nature Cell Biology.

[27] Sohni A, Tan K, Song HW, Burow D, de Rooij DG, Laurent L, Hsieh TC, Rabah R, Hammoud SS, Vicini E, Wilkinson MF (2019). The Neonatal and Adult Human Testis Defined at the Single-Cell Level. Cell Reports.

[28] O'Shaughnessy PJ, Fleming LM, Jackson G, Hochgeschwender U, Reed P, Baker PJ (2003). Adrenocorticotropic hormone directly stimulates testosterone production by the fetal and neonatal mouse testis. Endocrinology.

[29] Engels M, Span PN, Mitchell RT, Heuvel J, Marijnissen-van Zanten MA, van Herwaarden AE, Hulsbergen-van de Kaa CA, Oosterwijk E, Stikkelbroeck NM, Smith LB, Sweep F (2017). GATA transcription factors in testicular adrenal rest tumours. Endocr Connect.

[30] Lotfi CF, de Mendonca PO (2016). Comparative Effect of ACTH and Related Peptides on Proliferation and Growth of Rat Adrenal Gland. Frontiers in Endocrinology.

[31] McEwan PE, Vinson GP, Kenyon CJ (1999). Control of adrenal cell proliferation by AT1 receptors in response to angiotensin II and low-sodium diet. American Journal of Physiology.

[32] Combes-Moukhovsky ME, Kottler ML, Valensi P, Boudou P, Sibony M, Attali JR (1994). Gonadal and adrenal catheterization during adrenal suppression and gonadal stimulation in a patient with bilateral testicular tumors and congenital adrenal hyperplasia. Journal of Clinical Endocrinology and Metabolism.

[33] Bernichtein S, Alevizaki M, Huhtaniemi I (2008). Is the adrenal cortex a target for gonadotropins?. Trends in Endocrinology and Metabolism.

[34] Kolli V, da Cunha IW, Kim S, Iben JR, Mallappa A, Li T, Gaynor A, Coon SL, Quezado MM, Merke DP (2021). Morphologic and Molecular Characterization of Adrenals and Adrenal Rest Affected by Congenital Adrenal Hyperplasia. Frontiers in Endocrinology.

