# Supplementary figures and images for: Transcriptional comparison of Testicular Adrenal Rest Tumors with fetal and adult tissues
Source: Eur J Endocrinol. Author manuscript; Available in PMC 2022 Dec 9. (PMC7613903; doi:10.1530/EJE-22-0143)

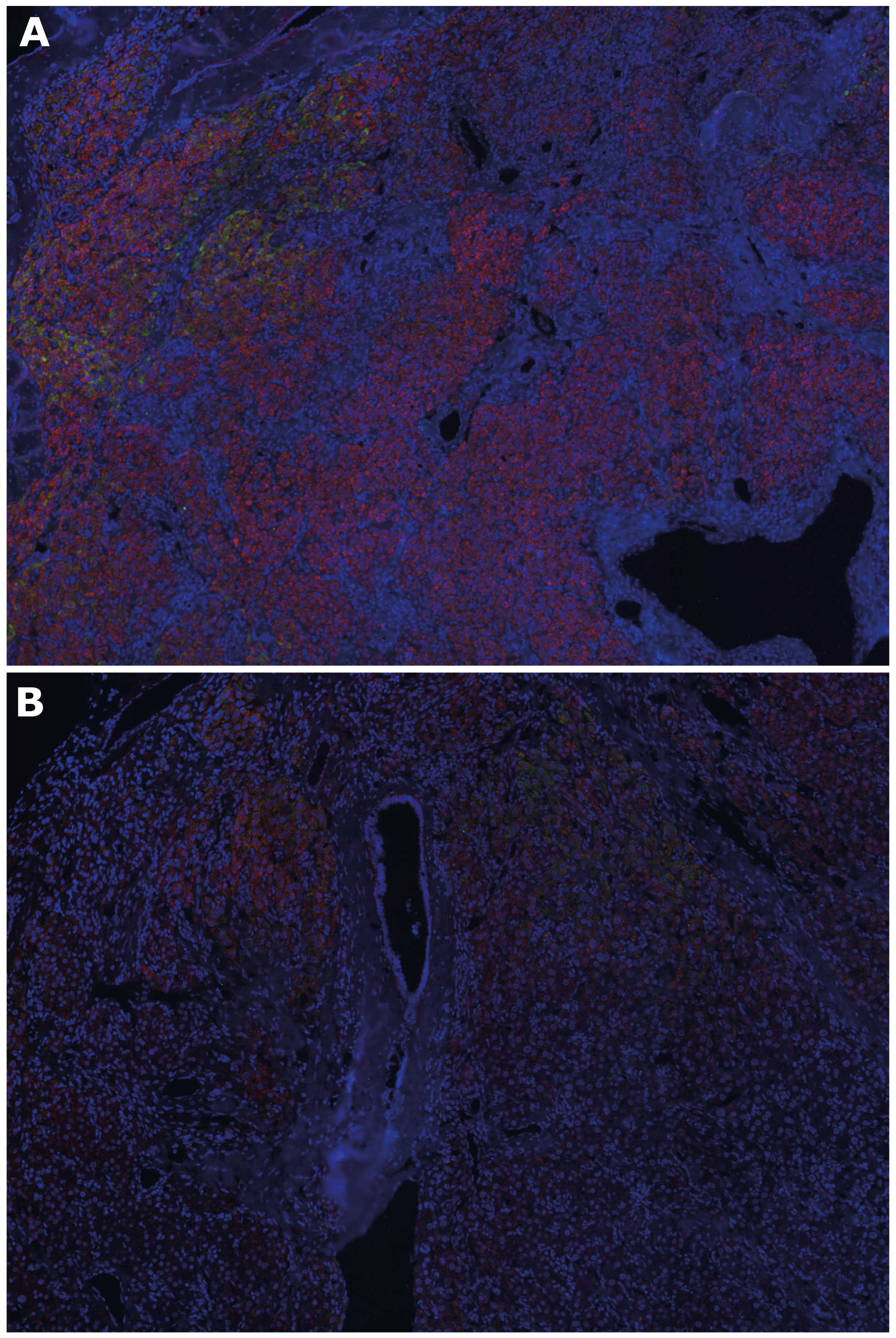

Supplement: Supplementary Figure 1 — Overview of two TART tissues (co-)expressing adrenal-specific 11β-hydroxylase (CYP11B1, red) and Leydig cell-specific 17β-hydroxysteroid dehydrogenase 3 (HSD17B3, green). Nuclei were stained with Hoechst (blue). [file EMS157771-supplement-Supplementary_Figure_1.tif]
